# Skeletal complications in patients with hemophilia: a single-center experience

**DOI:** 10.1186/s13018-023-04409-w

**Published:** 2023-11-29

**Authors:** Mohammadreza Bordbar, Razieh Beigipour, Mohammad Tahami, Omid reza Zekavat, Sezaneh Haghpanah, Reza Moshfeghinia

**Affiliations:** 1 Hematology Research Center, Shiraz University of Medical Sciences, Shiraz, Iran; 2 Pediatrics Department, Shiraz Medical School, Shiraz University of Medical Sciences, Shiraz, Iran; 3 Bone and Joint Disease Research Center, Shiraz University of Medical Sciences, Shiraz, Iran; 4 Student Research Committee, Shiraz University of Medical Sciences, Shiraz, Iran

**Keywords:** Hemophilia, Musculoskeletal complications, Arthropathy

## Abstract

**Background:**

Arthropathy is a common complication in patients with hemophilia. We examined the prevalence of this skeletal complication in patients with hemophilia who were registered at a Comprehensive Hemophilia Center in Shiraz, Southern Iran.

**Materials and Methods:**

In this cross-sectional study, an orthopedic specialist visited 448 patients and conducted screenings for skeletal complications. The assessment included evaluating the type of hemophilia, disease severity, treatment modality, the presence of inhibitors, and the identification of skeletal complications.

**Results:**

Ninety patients with hemophilia A, with a mean age (SD) of 31.6 (14.4) years, and 10 patients with hemophilia B, with a mean age of 30.5 (20.6) years, were assessed. The most frequently affected joints were the knee and ankle joints. In the univariate analysis, patients with severe disease were more likely to exhibit synovitis, a target joint, and bone disease compared to patients with non-severe disease. Additionally, a history of treated or active hepatitis and an annual bleeding rate showed significant associations with the target joint. In the multivariable logistic regression analysis, disease severity (OR 14.43, 95% CI 1.6–129.6) and a higher age at diagnosis (OR 1.06, 95% CI 1.00–1.13) increased the likelihood of developing osteoporosis. A history of hepatitis (OR 3.67, 95% CI 1.28–10.48) was identified as an independent risk factor for the target joint.

**Conclusion:**

Skeletal complications are a common occurrence in hemophilia. Regular consultations with orthopedic specialists, focusing on bleeding control and hepatitis prevention, are essential for reducing the impact of this debilitating complication.

## Introduction

Hemophilia Hemophilia is an inherited bleeding disorder that arises from a deficiency of coagulation factor (F)VIII in hemophilia A and Coagulation factor IX (FIX) in hemophilia B [1]. It is the most prevalent severe inherited coagulopathy globally, with an annual incidence rate of one in 5000 men in hemophilia A and 3.8 per 100,000 men in hemophilia B. The majority of cases are passed down through families with a documented history of the condition, but up to 55% of severe hemophilia A cases and 43% of severe hemophilia B cases result from non-hereditary factors due to new mutations [2].

Patients with hemophilia are prone to experiencing bleeding in various parts of the body, primarily in the musculoskeletal system [3, 4]. The persistent joint bleeding can give rise to a range of complications, including limb deformities, hypertrophy of the growth epiphyses, damage to the articular cartilage, asymmetrical hypertrophy, and ultimately joint destruction, a condition known as hemophilic arthropathy (HA) [3, 5, 6]. HA stands as one of the most prevalent complications associated with severe hemophilia [7]. Joint bleeding primarily manifests in the knees, ankles, and elbows, resulting in pain, deformities, and impaired joint functionality. Consequently, this has a significant impact on the quality of life for these patients [8].

The mechanism of HA is multifactorial and involves chronic or episodic synovitis, resulting in the destruction of cartilage, the formation of subchondral and bone cysts, and erosion and narrowing of joint space [9, 10]. The pathological mechanism of HA is intricate and encompasses characteristics of joint damage caused by inflammation, such as in rheumatoid arthritis (RA) and degenerative osteoarthritis (OA). Synovitis stands out as one of the most prominent pathologies in HA-afflicted joints. The synovium adheres to the inner surface of the joint capsule to maintain the intra-articular environment's equilibrium with the outside, giving rise to the principal components of synovial fluid [11, 12].

The primary factor behind synovial inflammation is widely regarded to be an elevation in iron load, leading to enhanced synthesis and proliferation of fibroblasts, ultimately resulting in synovium thickening [13, 14]. Hyaluronic acid (HA) is linked to chronic joint pain, particularly in multiple joints such as the knees, ankles, and elbows. Maintaining bone health is of utmost significance for individuals with HA [15]. Patients with HA commonly exhibit generalized osteoporosis and localized bone damage within their joints [16, 17].

Prophylaxis with coagulation factors, whether primary or secondary, has significantly diminished the frequency and intensity of HA. Nevertheless, this complication remains the most incapacitating challenge for individuals with severe diseases, particularly in resource-constrained countries [4].

Given the high prevalence of hemophilia and its associated complications within the Iranian population, coupled with a scarcity of data regarding musculoskeletal complications, our objective was to investigate and document these complications in a cohort of individuals with hemophilia in Shiraz, Southern Iran.

## Methods

### Study design

This single-center, cross-sectional study was conducted at a Comprehensive Hemophilia Center in Shiraz, Southern Iran, spanning from 2019 to 2021. The study aimed to include all registered patients with hemophilia A or B who had encountered skeletal or joint complications. Patients with congenital skeletal malformations were excluded from the study. A single orthopedic specialist examined 448 hemophilia patients, specifically those presenting musculoskeletal complications. Demographic information, including their current age, age at diagnosis, type and severity of hemophilia, treatment plan (on-demand or prophylaxis), annual bleeding rate (ABR), and hepatitis C infection status, was documented. The study also identified target joints, defined as joints that had experienced spontaneous bleeding more than three times in the last three months, and these joints underwent a thorough orthopedic examination and radiologic evaluation. To measure lumbar spine (L1–L4) and femoral neck bone mineral densitometry (BMD), the Hologic system dual-energy X-ray absorptiometry (DXA) (DiscoveryQDR, USA) was employed. Low bone mass (LBM) was defined as a Z-score of − 2 or lower, falling below the expected range according to the International Society for Clinical Densitometry (ISCD) definition [18]. The coefficient of variation was 0.5% for the lumbar spine and 1.5% for the femoral neck based on the two times BMD measurements taken on the same day from ten patients at our center, and the precision errors were determined using the root mean square method. Bone disease was considered present if the patients exhibited any signs of fractures, synovitis, osteoporosis, target joint, or osteoarthritis.

### Ethics

The data were collected anonymously. The research protocol adhered to the Declaration of Helsinki and was executed accordingly. Patients or their legal guardians were duly informed about the study, and a written consent form was signed. The study received approval and oversight from the Ethics Committee of Shiraz University of Medical Sciences in Shiraz, Iran, with the Ethics code IR.SUMS.MED.REC.1399.423.

### Analysis

The analysis of the data was conducted using IBM SPSS 23 statistical software. The normal distribution of data was assessed using the Kolmogorov–Smirnov test. Descriptive statistics were utilized to present the data, including the mean and standard deviation, or Interquartile range (IQR), along with frequency and percentage. Qualitative variables were assessed in various groups using the Chi-square test, while quantitative variables were compared using t tests. In cases where data did not follow a normal distribution, the Mann–Whitney test was employed. To identify independent factors influencing bone involvement, logistic regression analysis was carried out. A p value less than 0.05 was considered to be statistically significant.

## Results

### Descriptive findings

One hundred male individuals with an average (SD) age of 31.5 (15.1) years and an age range of 2–76 years were included. Ninety percent of the participants had hemophilia A. The demographic data are presented in Table 1. Patients with hemophilia A and B exhibited similarities in all clinical characteristics except for disease severity and target joints. Hemophilic arthropathy was observed in all patients, with varying degrees of severity. The majority of patients (93%) experienced involvement of multiple joints. Among those with single-joint arthropathy, knee joints were the most commonly affected. Two patients with hemophilia A were found to have high-titer (> 5 BU) neutralizing antibodies, while no inhibitory antibodies were detected in hemophilia B patients. Approximately 15% of hemophilia A patients and 40% of hemophilia B patients received prophylactic treatment with recombinant or plasma-derived factor concentrate.

**Table 1:** Demographic data and clinical information of participants

Variables	Haemophilia A (*n *= 90) Number (%)	Haemophilia B (*n *= 10) Number (%t)	*P* value
*Age at diagnosis (year)*			
< 1	56 (62.2)	4 (50)	–
1–5	16 (17.7)	2 (25)	
5–10	6 (6.6)	1 (12.5)	
> 10	12 (11.1)	1(12.5)	
Current age (Y) mean (SD)	31.6 (14.4)	30.5 (20.6)	0.82
*Severity of disease*			
Mild	16 (17.8)	1 (10)	0.027
Moderate	1 (1.1)	2 (20)	
Severe	73 (81.1)	7 (70)	
*Inhibitor*			
No	88 (97.8)	10 (100)	< 0.999
Yes	2 (2.2)	0 (0)	
*Annual bleeding*			
1–3	19 (21.1)	4 (40)	0.306
4–6	21 (23.3)	3 (30)	
7–12	45 (50)	2 (20)	
< 12	5 (5.6)	1 (10)	
*Treatment*			
Prophylaxis	14(15.6)	4(40)	0.077
On-demand	76(84.4)	6(60)	
*Number of joints involved*			
Single joint	6 (6.7)	1 (10)	0.481
Multiple joints	84 (93.3)	9 (90)	
*Target joint*			
Yes	39 (43.3)	0 (0)	0.006
No	51 (56.7)	10 (100)	
*Radiosynovectomy by 32P chromic phosphate*			
Yes	18 (20)	2 (20)	> 0.999
No	72 (80)	8 (80)	
*History of surgery*			
Yes	16 (17.8)	2 (20)	> 0.999
No	74 (82.2)	8 (80)	
*Fracture*			
Yes	6 (6.7)	0 (0)	> 0.999
No	84 (93.3)	10 (100)	
*Osteoporosis*			
Yes	35 (38.9)	4 (40)	> 0.999
No	55 (61.1)	6 (60)	
*Hepatitis C infection*			
Active			
Yes	14 (15.6)	0 (0)	0.349
No	76 (84.4)	10 (100)	
History			
Yes	26 (28.9)	0 (0)	0.060
No	64 (71.1)	10 (100)	
*Osteoarthritis*			
Yes	59(65.6)	5(50)	0.489
No	31(34.4)	5(50)	

### Factors affecting skeletal manifestations

The likelihood of bone disease, synovitis, and target joints was greater among patients with severe hemophilia than among non-severe patients. Over 43% of patients with hemophilia A and none of the hemophilia B patients developed target joints (*p *< 0.001). The treatment plan (prophylaxis vs. on-demand) had no impact on the probability of bone disease in the examined patients.

Patients who had previously or currently had a hepatitis C infection were observed to be at a higher susceptibility to develop target joints. Furthermore, those individuals with an ABR exceeding three times per year exhibited a higher frequency of target joint development (*P *= 0.016). Patients with a higher ABR also faced a significantly increased risk of bone disease (*P *= 0.003) (Table 2).

**Table 2:** Comparison of factors affecting skeletal manifestations in patients with hemophilia

Variables		Skeletal manifestations number (%)					
	Subgroups	Fracture	Synovitis	Osteoporosis	Target joint	Osteoarthritis	Bone disease
Severity of disease	Severe	6 (7.5)	16 (20)	35 (43.8)	37 (46.3)	53 (66.3)	68 (85)
	Non-severe	0 (0)	0 (0)	4 (20)	2 (10)	11 (55)	12 (60)
	*P* value	0.59	0.03	0.07	< 0.001	0.43	0.02
Type of hemophilia	A	6 (6.7)	14 (15.6)	35 (38.9)	39 (43.3)	59 (65.6)	74 (82.2)
	B	0 (0)	2 (20)	4 (40)	0 (0)	5 (50)	6 (60)
	*P* value	> 0.99	0.66	> 0.99	< 0.001	0.48	0.11
Treatment	On-demand	6 (7.3)	11 (13.4)	30 (36.6)	35 (42.7)	55 (67.1)	68 (82.9)
	prophylaxis	0 (0)	5 (27.8)	9 (50)	4 (22.2)	9 (50)	12 (66.7)
	*P* value	0.588	0.158	0.301	0.120	0.186	0.189
History of hepatitis C infection	Yes	4 (5.4)	13 (17.6)	29 (39.2)	21 (28.4)	45 (60.8)	56 (75.7)
	No	2 (7.7)	3 (11.5)	10 (38.5)	18 (69.2)	19 (73.1)	24 (92.3)
	*P* value	0.649	0.552	> 0.999	< 0.001	0.344	0.089
Active hepatitis C infection	Yes	6 (7)	13 (15.1)	36 (41.9)	29 (33.7)	55 (64)	68 (79.1)
	No	0 (0)	3 (21.4)	3 (21.4)	10 (71.4)	9 (64.3)	12 (85.7)
	*P* value	0.591	0.693	0.237	0.015	> 0.999	0.730
Annual bleeding rate	1–3	2 (8.7)	1 (4.3)	10 (43.5)	4 (17.4)	12 (52.2)	13 (56.5)
	4–6	1 (4.2)	4 (16.7)	10 (41.7)	8 (33.3)	17 (70.8)	22 (91.7)
	> 6	3 (5.7)	11 (20.8)	19 (35.8)	27 (50.9)	35 (66)	45 (84.9)
	*P* value	0.740	0.221	0.795	0.019	0.357	0.004
Annual bleeding rate	1–3	2 (8.7)	1 (4.3)	10 (43.5)	4 (17.4)	12 (52.2)	13 (56.5)
	> 3	4 (5.2)	15 (19.5)	29 (37.7)	35 (45.5)	52 (67.5)	67 (87)
	*P* value	0.619	0.109	0.634	0.016	0.218	0.003

In the realm of multivariate analysis, individuals with a prior history of hepatitis C infection exhibited a risk of bone disease that was more than threefold higher (OR 3.18, 95% CI 0.98–10.31, *P *= 0.053). Likewise, a history of hepatitis C infection was found to elevate the likelihood of developing target joints, with an odds ratio of 3.67 (95% CI 1.28–10.48, *P *= 0.015). Furthermore, patients with severe hemophilia faced a nearly tenfold increased risk of developing target joints (OR 10.12, 95% CI 0.93–109.3, *P *= 0.057). Osteoporosis exhibited a prevalence that was over 14 times greater in patients with severe hemophilia when compared to their non-severe counterparts (OR 14.43, 95% CI 1.6–129.6, *P *= 0.017). Additionally, a delayed diagnosis of hemophilia at a later age was linked to an augmented risk of osteoporosis, with a 6% increase for each year of delay (OR 1.06, 95% CI 1.00–1.13, *P *= 0.051) (see Table 3).

**Table 3:** Multivariable logistic regression analysis of covariates associated with various bone diseases in patients with hemophilia

Variables	Skeletal complications					
	Bone disease		Osteoporosis		Target joint	
	OR (95% CI)	*P* value	OR (95% CI)	*P* value	OR (95% CI)	*P* value
Treatment^*^	2.54 (0.64–10.02)	0.182	0.61 (0.19–1.96)	0.408	1.93 (0.49–7.58)	0.341
Age at diagnosis	0.99 (0.95–1.04)	0.944	1.06 (1.00–1.13)	0.051	1.02 (0.95–1.10)	0.436
Annual bleeding rate^**^	2.10 (0.40–10.97)	0.378	0.69 (0.21–2.22)	0.540	2.00 (0.50–8.06)	0.325
Severity of hemophilia^***^	2.94 (0.68–12.68)	0.146	14.43 (1.60–129.6)	0.017	10.12 (0.93–109.3)	0.057
History of hepatitis C infection	3.18 (0.98–10.31)	0.053	0.94 (0.34–2.55)	0.909	3.67 (1.28–10.48)	0.015

*OR* Odds ratio, *CI* Confidence interval

*Prophylaxis vs. on-demand factor replacement therapy

** > 3 bleeding/year versus 1–3 bleeding/year

***Severe hemophilia versus non-severe hemophilia

## Discussion

Hemophilic arthropathy is a common complication in individuals with hemophilia. While prophylactic factor replacement has significantly reduced the number of joint bleeding episodes, hemophilic arthropathy still affects 25–30% of those with hemophilia, as reported in previous studies [19]. In our registered patient population with hemophilia, we observed a rate of 22.3% (100 out of 448). Recent evidence indicates that the pathophysiology of hemophilic arthropathy involves complex inflammatory and immunologic mechanisms. Occurrence of synovitis and hemophilic arthropathy is directly associated with the severity of the illness and the annual bleeding rate (ABR) [20]. We also noted a higher prevalence of synovitis and target joints in patients with severe hemophilia. Moreover, an ABR exceeding three times per year was linked to a higher incidence of bone diseases, including target joints, in our patient population.

Currently, primary prophylaxis remains the gold standard for patients with severe hemophilia. It plays a crucial role in averting joint damage by mitigating the frequency and severity of hemarthrosis. Around 15% of our hemophilia A patients and 40% of hemophilia B patients are administered a prophylactic factor replacement regimen. While the incidence of target joint development was lower in the prophylactic treatment group compared to the on-demand approach, this disparity did not reach statistical significance. One plausible rationale is that some of these patients were initiated on prophylaxis after experiencing joint damage, in an effort to prevent further bleeding. It is essential to bear in mind that hemarthrosis can still manifest even with primary prophylaxis using factor concentrates [21].

Notably, none of our patients with hemophilia B experienced target joints. It has been previously noted that the occurrence and intensity of arthropathy in patients with hemophilia B are lower compared to hemophilia A, even when accounting for age and illness severity [6].

We noted a significant prevalence of low bone mass among our patients with hemophilia, approximately 40% in both hemophilia A and B. Certain researchers have documented even higher rates, reaching up to 70% (comprising 43% osteopenia and 27% osteoporosis as per the WHO classification) [22]. The results of two meta-analyses confirm that there is a significant increase in low BMD in the lumbar spines and femoral neck of patients with hemophilia when compared to controls [23]. We employed the ISCD criteria to define low bone mass, which solely characterizes low bone mass as Z-score ≤  − 2, disregarding osteopenia. Multiple risk factors for low bone mass in hemophilia have been suggested, encompassing vitamin D deficiency, smoking, low body mass index (BMI), alcoholism, and physical inactivity [24]. We have noted that the risk of osteoporosis is markedly higher in severe hemophilia patients compared to those with non-severe hemophilia (OR 14.4, 95% CI 1.6–129.6). Furthermore, individuals diagnosed later in life faced an increased risk of low bone mass, with rates being 6% higher for every one-year delay in diagnosis. Our prior study also indicated that low bone mass was linked to disease severity, low BMI, and hepatitis C infection in patients with hemophilia [25]. There is also a novel concept suggesting that Factor VIII (FVIII) has an impact on bone health beyond its role in the coagulation system. It is theorized that thrombin contributes to bone remodeling by impeding osteoclast differentiation and promoting osteoblast proliferation. Consequently, a deficiency in FVIII results in decreased bone mass due to diminished thrombin production. Furthermore, the FVIII and Von-Willebrand factor complex can impede bone resorption induced by receptor activator of nuclear factor kappa beta (RANKL) [24].

Approximately 30% of our hemophilia A patients had a history of hepatitis C infection, with half of them currently experiencing an active infection. It is estimated that 40–90% of hemophilia patients suffer from chronic hepatitis C infection, primarily stemming from the receipt of contaminated blood products in the early 1980s [26]. While the primary complications of hepatitis C infection are typically liver cirrhosis and hepatocellular carcinoma, there appears to be an increased occurrence of musculoskeletal complications, such as osteoporosis and arthropathy [27]. We observed a significantly increased risk of bone diseases and target joints (more than 3 times higher) in our patients with a history of hepatitis C infection. This association has not been previously reported. The inflammatory response triggered by the viral infection and subsequent cytokine release may contribute to synovial damage, ultimately leading to hemophilic arthropathy.

The study was constrained by the relatively small sample size and the unavailability of MRI images for diagnosing subclinical synovitis. Nonetheless, we have reported, for the first time in the literature, an association between hepatitis C infection and HA. This discovery should be validated in future multicenter studies with a larger sample size. Implementing annual screening for viral infections and timely management may aid in averting the progression of musculoskeletal complications in hemophilia patients.

## Conclusion

Musculoskeletal complications are commonly observed in individuals with severe hemophilia. Preventing recurrent bleeding through appropriate factor replacement therapy is a critical measure to prevent the development of target joints and hemophilic arthropathy. Timely diagnosis and management of hepatitis C infection is strongly advised.
